# Use of ER/PR/HER2 subtypes in conjunction with the 2007 St Gallen Consensus Statement for early breast cancer

**DOI:** 10.1186/1471-2407-10-228

**Published:** 2010-05-21

**Authors:** Katrina Bauer, Carol Parise, Vincent Caggiano

**Affiliations:** 1 California Cancer Registry, California Department of Public Health, Public Health Institute, Sacramento CA, USA; 2 Sutter Institute for Medical Research, 2801 Capitol Ave Suite 400, Sacramento CA, USA

## Abstract

**Background:**

The 2007 St Gallen international expert consensus statement describes three risk categories and provides recommendations for treatment of early breast cancer. The set of recommendations on how to best treat primary breast cancer is recognized and used by clinicians worldwide. We now examine the variability of five-year survival of the 2007 St Gallen Risk Classifications utilizing the ER/PR/HER2 subtypes.

**Methods:**

Using the population-based California Cancer Registry, 114,786 incident cases of Stages 1-3 invasive breast cancer diagnosed between 2000 and 2006 were identified. Cases were assigned to Low, Intermediate, or High Risk categories. Five-year-relative survival was computed for the three St Gallen risk categories and for the ER/PR/HER2 subtypes for further differentiation.

**Results and Discussion:**

There were 9,124 (13%) cases classified as Low Risk, 44,234 (65%) cases as Intermediate Risk, and 14,340 (21%) as High Risk. Within the Intermediate Risk group, 33,735 (76%) were node-negative (Intermediate Risk 2) and 10,499 (24%) were node-positive (Intermediate Risk 3). For the High Risk group, 6,149 (43%) had 1 to 3 positive axillary lymph nodes (High Risk 4) and 8,191 (57%) had four or more positive lymph nodes (High Risk 5).

Using five-year relative survival as the principal criterion, we found the following: a) There was very little difference between the Low Risk and Intermediate Risk categories; b) Use of the ER/PR/HER2 subtypes within the Intermediate and High Risk categories separated each into a group with better five-year survival (ER-positive) and a group with worse survival (ER-negative), irrespective of HER2-status; c) The heterogeneity of the High Risk category was most evident when one examined the ER/PR/HER2 subtypes with four or more positive axillary lymph nodes; (d) HER2-positivity did not always translate to worse survival, as noted when one compared the triple positive subtype (ER+/PR+/HER2+) to the triple negative subtype (ER-/PR-/HER2-); and (e) ER-negativity appeared to be a stronger predictor of poor survival than HER2-positivity.

**Conclusion:**

The use of ER/PR/HER2 subtype highlights the marked heterogeneity of the Intermediate and High Risk categories of the 2007 St Gallen statements. The use of ER/PR/HER2 subtypes and correlation with molecular classification of breast cancer is recommended.

## Background

The 2007 St Gallen international expert consensus statement described three risk categories and provided recommendations for treatment of early breast cancer [1]. Since the first publication of the consensus statements in 1988 [2], now updated every two years, the set of recommendations on how to best treat primary breast cancer is recognized and used by clinicians worldwide.

Molecular classification is rapidly becoming the gold standard for complete characterization of breast cancer and the underlying technology has already led to generation of gene-profiling models to predict outcomes [3-5]. Despite these remarkable achievements, clinicians still rely on traditional tumor marker analysis for treatment decisions. We recently described the distribution and five-year survival of the subtypes of breast cancer based on estrogen receptor (ER), progesterone receptor (PR), and human epidermal growth factor receptor 2 (HER2) [6,7], and found wide variation in survival especially among the HER2-positive group of patients. We now examine the variability of five-year survival of the 2007 St Gallen Risk Classifications utilizing the ER/PR/HER2 subtypes.

## Methods

Breast cancer cases used in these analyses were identified using the population-based California Cancer Registry (CCR). Cases are reported to the Cancer Surveillance Section of the California Department of Public Health from hospitals and any other facilities providing care or therapy to cancer patients residing in California [8].

For the current study, we identified 114,786 first primary cases of invasive breast cancer (ICDO-3 sites C50.0-C50.9) [9] diagnosed between January 1, 2000 and December 31, 2006, and reported to the CCR as of December 2008. This study period was selected because it is prior to the era when trastuzumab was routinely administered to women with early breast cancer that overexpressed HER2. Cases diagnosed outside of California, at autopsy, or from death certificates were excluded.

The CCR requires the collection of tumor marker information from the medical record on the status of ER and PR for breast cancers diagnosed on or after January 1, 1990 and HER2 for breast cancers diagnosed on or after January 1, 1999. ER and PR status are recorded according to the pathologist's interpretation of assays. A tumor is considered to be ER negative and PR negative if less than 5% of tumor cell nuclei are immunoperoxidase positive in immunohistochemistry (IHC) assays. ER and PR status may also have been determined by examining cytosol protein (ER negative or PR negative if there are fewer than 3 or 5 fmol/mg of cytosol protein, respectively) HER2 was assessed through IHC or fluorescence in situ hybridization (FISH). IHC is scored on a qualitative scale based on staining intensity: 0 and 1+ are negative, 2+ is borderline, and 3+ is positive. FISH is scored on a quantitative scale: less than 2 copies of the HER2 gene is negative and 2 or more copies is positive. Cases with complete tumor marker data were used in this study and were categorized into one of the eight distinct subtypes based on ER/PR/HER2 status of the tumor. Cases with unknown or borderline tumor marker status were excluded from these analyses [8].

Stage at diagnosis was collected from the patient's medical record and coded according to the American Joint Commission on Cancer (AJCC) Cancer Staging Manual 6^th ^edition [10]. The CCR collected Surveillance Epidemiology and End Results (SEER) Extent of Disease (EOD) for breast cancer cases diagnosed from 1988 through December 2003 [11], and in 2004, began collecting Collaborative Staging data items [12]. EOD was converted to AJCC stage at diagnosis using SEER guidelines [13]. For these analyses, stage IV and cases with unknown stage were omitted. Tumor grade was collected from the medical record and coded according to ICDO-3 [9].

Treatment information available from the registry abstract was recorded as one of four possibilities: a) chemotherapy without endocrine therapy; b) endocrine therapy without chemotherapy; c) chemotherapy and endocrine therapy; d) no chemotherapy or endocrine therapy or unknown. Information about the use of specific anti-HER2 therapy was not available.

Cases were assigned to Low, Intermediate, or High Risk categories according to published criteria [1] with one exception. The extent of peritumoral vascular invasion was not available from the CCR's database and could not be utilized for determining the St Gallen risk stratification.

Counts and 5-year relative cumulative survival were calculated using SEER*Stat 6.1.4. The actuarial method was used for relative survival calculations. Five-year-relative survival was computed for the three St Gallen risk categories, and also for the ER/PR/HER2 subtypes to further differentiate the risk categories. Differences between survival curves were compared using the Z- test for comparison of relative survival rates [14].

## Results

There were 114,786 incident cases of Stages 1-3 invasive breast cancer diagnosed between 2000 and 2006. After exclusion of 38,418 cases with at least one missing tumor marker there were 76,368 cases. An additional 8,670 were eliminated because they lacked age, size of tumor, stage, or grade resulting in 67,698 (59%) cases available for survival analysis (Table 1). Table 2 shows the clinicopathologic breakdown of the cases included in the analysis.

**Table 1:** Summary of cases included in the analysis.

Cases with stages 1-3 1^st ^primary invasive breast cancer 2000-2006	114,786
Cases missing at least one tumor marker	38,418
Cases missing age, tumor size, stage, or grade	8,670
**Total included in the analysis**	**67,698**

**Table 2:** Demographic and clinicopathologic characteristics for stages 1-3 first primary invasive breast cancer, 2000-2006.

	n	%
Node Status		
Node Negative	42,859	63.3%
1-3 Nodes Positive	16,648	24.6%
>3 Nodes Positive	8,191	12.1%
**Stage**		
I	31,721	46.9%
II	28,952	42.8%
III	7,025	10.4%

**Grade**		
Well differentiated; Grade I	14,813	21.9%
Moderately differentiated; Grade II	28,406	42.0%
Poorly differentiated; Grade III	23,191	34.3%
Undifferentiated; anaplastic; Grade IV	1,288	1.9%

**ER/PR/HER2 Status**		
+/+/+	7,343	10.8%
+/+/-	36,906	54.5%
+/-/+	2,258	3.3%
+/-/-	6,305	9.3%
-/+/+	319	0.5%
-/-/+	4,805	7.1%
-/+/-	652	1.0%
-/-/-	9,110	13.5%

**Tumor Size**		
≤2 cm	42,043	62.1%
> 2 cm	25,655	37.9%

**Race/Ethnicity**		
Non-Hispanic White	47,016	69.4%
Non-Hispanic Black	3,606	5.3%
Hispanic	10,134	15.0%
Asian/Pacific Islander	6,942	10.3%

**Age**		
≤45	11,755	17.4%
46-69	40,058	59.2%
≥70	15,885	23.5%

**Total**	**67,698**	

**Figure 1:**
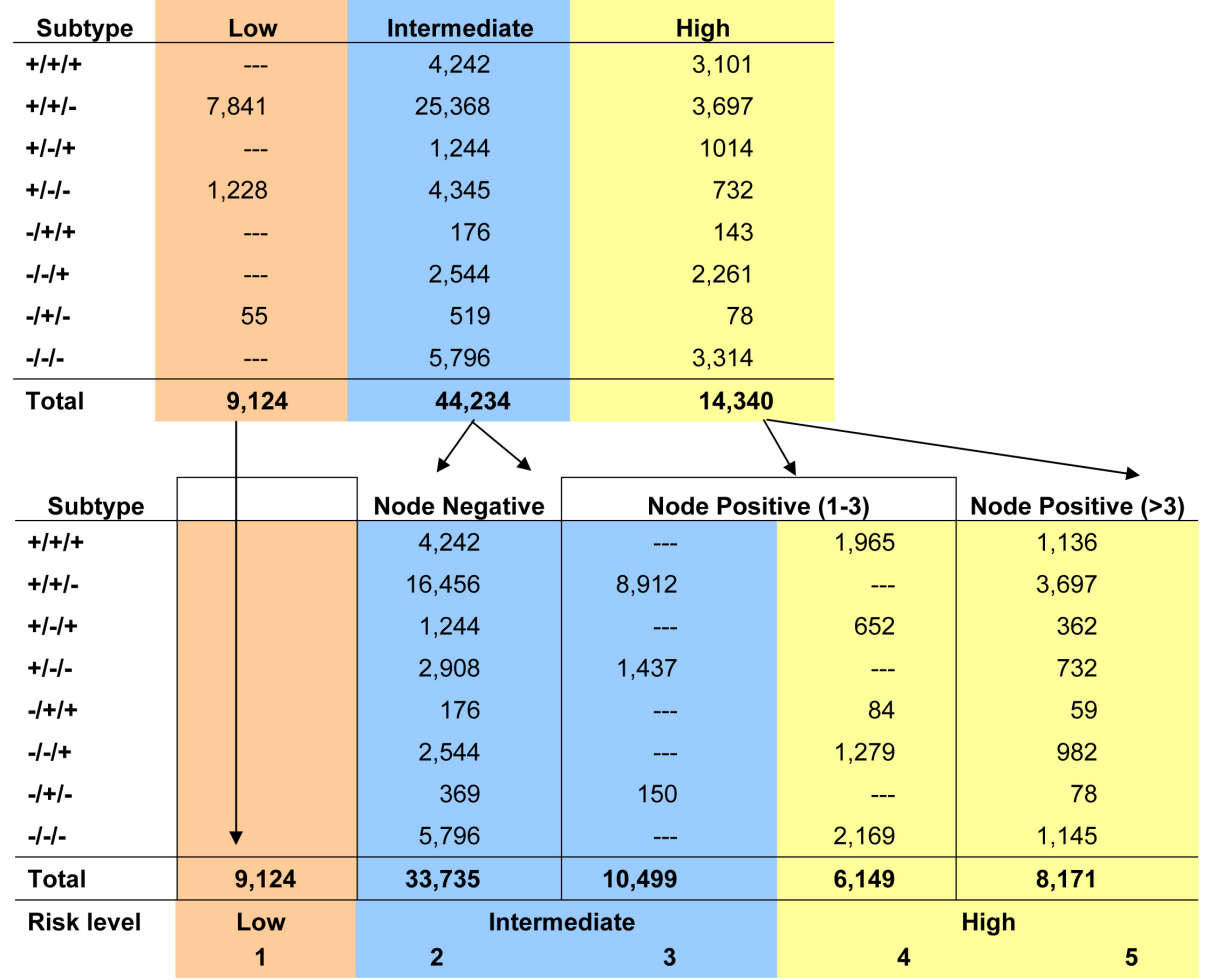
**Stratification of the ER/PR/HER2 subtypes within the St Gallen risk categories**.

The distribution of the ER/PR/HER2 subtypes within each risk category is shown in Table 3 and Figure 1. There were 9,124 (13%) cases classified as Low Risk, 44,234 (65%) cases as Intermediate Risk, and 14,340 (21%) as High Risk. Within the Intermediate Risk group, 33,735 (76%) were node-negative (Intermediate Risk 2) and 10,499 (24%) were node-positive (Intermediate Risk 3). For the High Risk group, 6,149 (43%) had 1 to 3 positive axillary lymph nodes (High Risk 4) and 8,191 (57%) had four or more positive lymph nodes (High Risk 5).

**Table 3:** Distribution of the ER/PR/HER2 subtypes by St Gallen risk category.

	Low Risk	Intermediate Risk 2	Intermediate Risk 3	High Risk 4	High Risk 5	n	%
**+/+/+**	0	4,242	0	1,965	1,136	**7,343**	**10.8**
**+/+/-**	7,841	16,456	8,912	0	3,697	**36,906**	**54.5**
**+/-/+**	0	1,244	0	652	362	**2,258**	**3.3**
**+/-/-**	1,228	2,908	1,437	0	732	**6,305**	**9.3**
**-/+/+**	0	176	0	84	59	**319**	**0.5**
**-/-/+**	0	2,544	0	1,279	982	**4,805**	**7.1**
**-/+/-**	55	369	150	0	78	**652**	**1.0**
**-/-/-**	0	5,796	0	2,169	1,145	**9,110**	**13.5**
**n**	**9,124**	**33,735**	**10,499**	**6,149**	**8,191**	**67,698**	
**%**	**13.5**	**49.8**	**15.5**	**9.1**	**12.1**		

**Figure 2:**
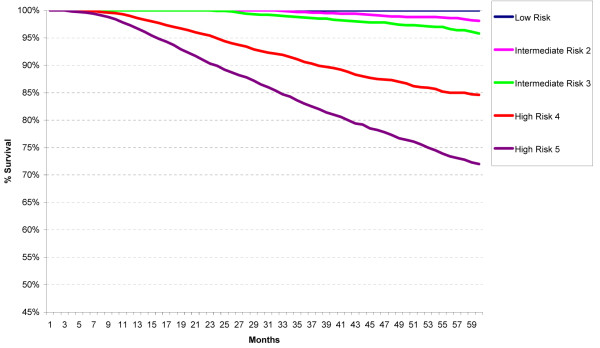
**Five-year relative survival of low, intermediate, and high risk St Gallen categories for first primary invasive breast cancers in California, 2000-2006**.

Five-year relative survival curves revealed a statistically significant difference between the Low and both Intermediate Risk categories (p < 0.001) (Figure 2). There was no statistically significant difference between the two Intermediate risk categories. The High Risk category is clearly separated from the Low and Intermediate Risk categories, and those with four or more positive lymph nodes (High Risk 5) have worse survival than those with 1 to 3 positive lymph nodes (High Risk 4) (p < 0.001).

**Figure 3:**
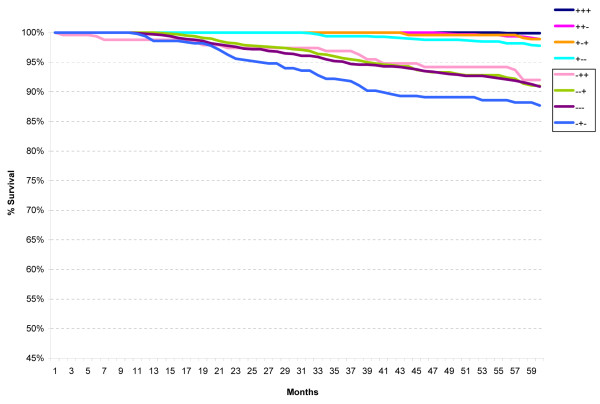
**Five-year relative survival for all cases within the St Gallen intermediate risk group according to the ER/PR/HER2 subtypes**.

**Figure 4:**
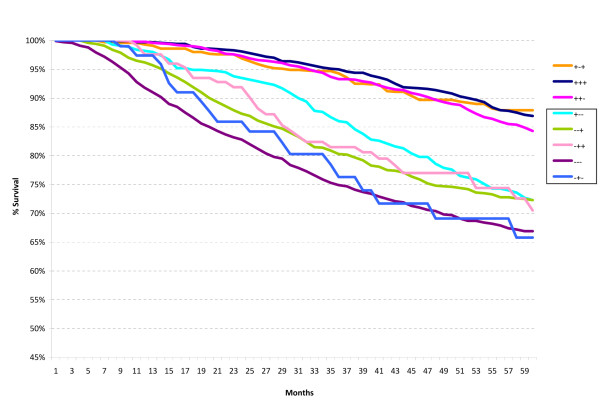
**Five-year relative survival for all cases within the St Gallen high risk group according to the ER/PR/HER2 subtypes**.

When the St Gallen Risk categories were analyzed according to ER/PR/HER2 subtypes, no difference in survival was seen in the Low Risk group (not shown) but distinct differences were noted in the Intermediate Risk (Figure 3) and High Risk (Figure 4) categories. All ER-positive subtypes within the Intermediate Risk group, regardless of HER2 status, had excellent five-year relative survival (95% or better), whereas all ER-negative subtypes had worse survival.

**Figure 5:**
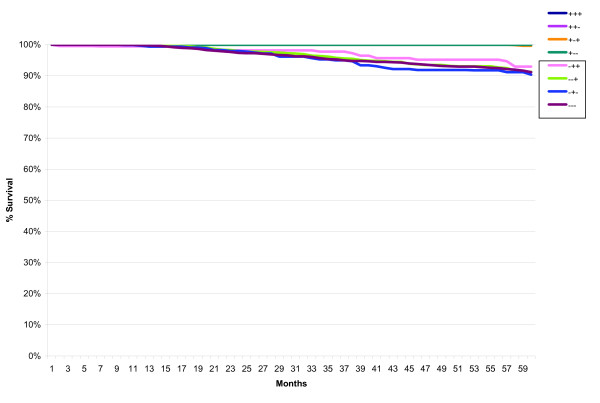
**Five-year relative survival for node-negative cases within the St Gallen intermediate risk group (intermediate risk 2) according to the ER/PR/HER2 subtypes**.

**Figure 6:**
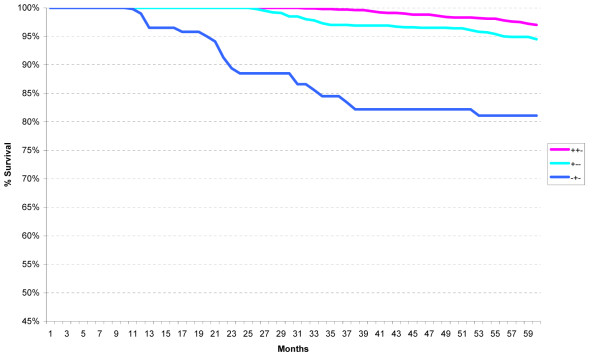
**Five-year relative survival for node-positive cases within the St Gallen intermediate risk group (intermediate risk 3) according to the ER/PR/HER2 subtypes**.

For the High Risk group, a similar ER-positive pattern was noted except for the ER+/PR-/HER2- subtype. When the Intermediate and High Risk categories were analyzed according to axillary lymph node status as well as ER/PR/HER2 status, the ER+/PR-/HER2+ and ER+/PR-/HER2- subtypes had equally excellent five-year relative survival within the node-negative group (Intermediate Risk 2) (p = 0.234), and the ER+/PR+/HER2- and ER+/PR-/HER2- subtypes had significantly better survival than the ER-/PR+/HER2- subtype within the node-positive group (Intermediate Risk 3), as seen in Figures 5 and 6 (p < 0.001 for both comparisons).

**Figure 7:**
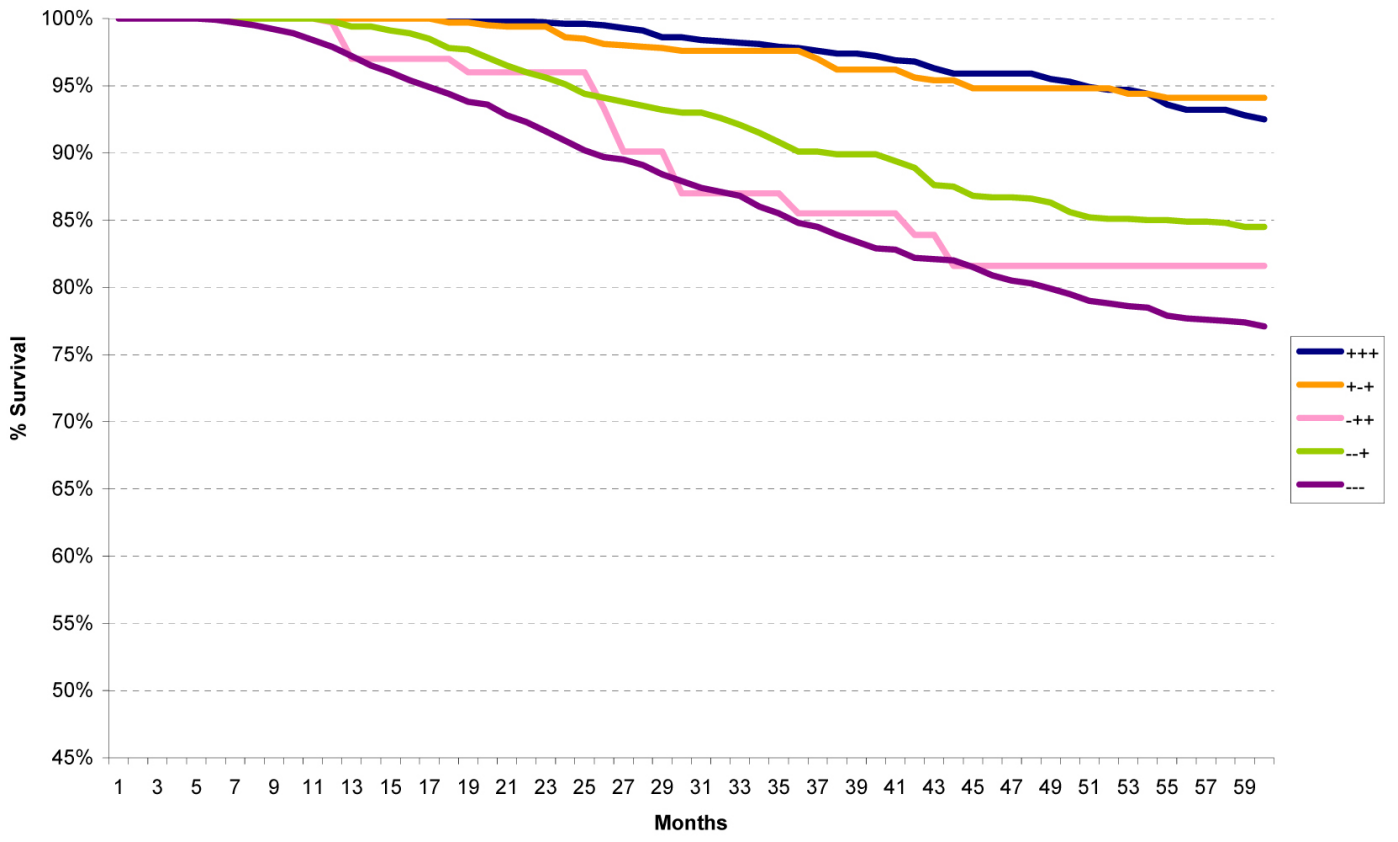
**Five-year relative survival for node positive cases with 1-3 lymph nodes within the St Gallen high risk group (high risk 4) according to the ER/PR/HER2 subtypes**.

**Figure 8:**
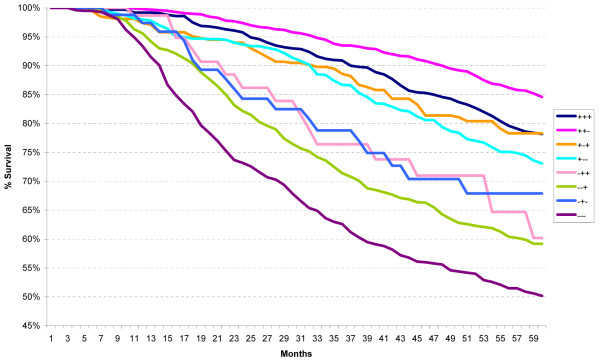
**Five-year relative survival for node positive cases with >3 lymph nodes within the St Gallen high risk group (high risk 5) according to the ER/PR/HER2 subtypes**.

Within the High Risk category characterized by 1 to 3 positive nodes (High Risk 4), the ER+/PR+/HER2+ and ER+/PR-/HER2+ subtypes had excellent five-year survival whereas the remaining subtypes, all ER-negative, had significantly worse survival (p < 0.02) (Figure 7). For those High Risk patients with four or more positive lymph nodes (High Risk 5), the best survival was seen in the ER+/PR+/HER2+ subtype and the worst survival in the ER-/PR-/HER2- subtype (Figure 8) (p < 0.001).

Treatment information is summarized in Table 4. Of the 4,717 cases within the Low Risk category known to have received some form of therapy, 4,019 (85%) received only endocrine treatment (ET) and 698 (7%) received chemotherapy and ET. For the Intermediate Risk category, 18,908 (66%) received chemotherapy with or without ET, and 9,629 (34%) received only ET. Within the High Risk category, 11,016 (94%) received chemotherapy with or without ET, and 671 (6%) received only ET.

**Table 4:** Differences in use of chemotherapy and endocrine therapy (ET) among the St Gallen risk categories.

	Low	Intermediate	High
Chemotherapy without ET	349	12,429	8,007
ET without chemotherapy	4,019	9,629	671
Chemotherapy + ET	349	6,479	3,009
None or Unknown	4,407	15,697	2,653

**Total**	**9,124**	**44,234**	**14,340**

## Discussion

Clinicians have a variety of resources and guidelines to assist in treatment decisions for early breast cancer patients [15]. Adjuvant! Online [16], the guidelines of the National Comprehensive Cancer Network [17], the 21-gene recurrence score [4], and the 70-gene expression assay [3], are well-known to most oncologists. The St Gallen Consensus Statements, however, remain a valuable tool, especially in Europe, perhaps because of its simplicity and ease of risk determination. Successive St Gallen conferences since 1988 have produced treatment guidance based on available evidence and expert opinion for the therapy of early breast cancer patients outside clinical trials. Various iterations of the St Gallen Consensus recommendations have been validated and compliance with recommendations for systemic therapy has been shown to improve survival of women with node-negative breast cancer, refinements have been suggested [18-24] and some shortcomings have been described [25,26].

Our initial CCR investigations of triple negative breast cancer [27,28] prompted us to examine all newly diagnosed breast cancer patients using ER/PR/HER2 subtype as a surrogate for the molecular classification [6,7]. We were struck by the wide variation in five-year survival based on ER/PR/HER2, the marked heterogeneity of HER2-positive cancers, and the excellent survival of patients with ER-positive cancers irrespective of HER2 status. Indeed, we found the ER-positive/PR-positive/HER2-positive subset of patients to have a five-year relative survival of 91.3% whereas the ER-negative/PR-negative/HER2-positive subset to have a 75.9% survival [7]. Clearly, not all HER2-positive breast cancers are bad actors. We therefore decided to explore this more fully and determine the ER/PR/HER2 subtypes within the context of the St Gallen risk categories.

The Intermediate Risk is the largest of the three risk categories defined in the 2007 St Gallen statement, constituting 65.3% of all patients, the Low Risk the smallest (13.4%) and the High Risk next at 21.3%. Based solely on five-year relative survival, there is not much difference between Low and Intermediate Risk, but clear separation of the High Risk category is noted.

Use of the ER/PR/HER2 subtypes clearly separates the Intermediate Risk category into two distinct groups, one with better five-year survival (all ER-positive) and one with worse survival (all ER-negative). This separation persists for the node-negative subgroup (Intermediate Risk 2) as well as the node-positive subgroup (Intermediate Risk 3), although in this latter subgroup the ER-/PR+/HER2- subtype is rather small. The existence of the ER-/PR+ subtypes is controversial [29,30] although some believe it is a distinctive subtype with a poor prognosis [31]. Our results, while not definitive in any sense, would tend to agree with this latter interpretation.

Marked variation in five-year survival is noted within the High Risk category, both for the subgroups with 1-3 positive axillary lymph nodes (High Risk 4) and >3 positive axillary lymph nodes (High Risk 5). The widest variation in five-year survival is seen for this latter subgroup, ranging from the triple positive subtype (ER+/PR+/HER2+) at 83% to the triple negative subtype (ER-/PR-/HER2-) at 48%. Clearly, there are different levels of risk. Once again, ER-positive subtypes have better survival than ER-negative subtypes, regardless of HER2 status.

That the 2007 St Gallen risk categories are useful for therapy guidance is evident from the limited treatment analysis, with 85% within Low Risk category receiving only ET, and 94% within High Risk category receiving chemotherapy with or without ET. However, our study has shown that, using five-year relative survival as the single principal criterion, (a) there is very little difference between Low Risk and Intermediate Risk categories, (b) use of the ER/PR/HER2 subtypes within the Intermediate and High Risk categories separates each into a group with better five-year survival (ER-positive) and a group with worse survival (ER-negative), irrespective of HER2-status, (c) the heterogeneity of the High Risk category is most evident when one examines the ER/PR/HER2 subtypes with four or more positive axillary lymph nodes, (d) HER2-positivity, per se, does not always translate to a worse survival, as noted when one compares the triple positive subtype (ER+/PR+/HER2+) to the triple negative subtype (ER-/PR-/HER2-), and (e) ER-negativity appears to be a stronger predictor of poor survival than HER2-positivity.

The 2009 St Gallen conference proposed "a radically different treatment selection algorithm for the management of early breast cancer" and abandoned the three risk categories [32]. Instead, the threshold for indication of each systemic treatment modality was outlined, and two situations were recognized in which the decision to use adjuvant chemotherapy was relatively clear-cut, i.e., for patients with triple negative breast cancer and for patients with HER2-positive breast cancer. The present study once again confirms the poor survival of the triple negative subtype and the heterogeneity of the HER2-positive group of patients [7,27]. Although combined endocrine therapy in addition to anti-HER2 therapy without chemotherapy in strongly ER-positive, HER2-positive patients is logical but unproven [32], our survival results for the HER2-positive subtypes within the Intermediate and High Risk categories suggest an opportunity for just such a randomized clinical trial utilizing traditional clinicopathological factors, tumor markers, and gene expression profiling assays.

In a departure from the previous conference, the panel of the 2009 St Gallen meeting also supported the use of a validated multigene-profiling assay, if readily available, as an adjunct to high quality phenotyping of breast cancer in cases in which the indication for adjuvant chemotherapy remains uncertain. We are in agreement with this recommendation, and further urge use of the ER/PR/HER2 subtype or phenotype expression as a surrogate, albeit imperfect, for the molecular classification of breast cancer [33,34]. Correlation of the ER/PR/HER2 subtypes with gene-profiling assays makes good clinical sense. We suggest avoidance of the phrase "ER and/or PR positive".

Although we studied a large, racially diverse group of breast cancer patients, we recognize the limitations of this type of population-based registry investigation. Histologic grading of tumors, as well as tests for ER, PR, and HER2 were performed by a wide variety of laboratories without central review. Only 59% of the original cohort of patients was found to have complete clinicopathological factors. Of the 38,418 cases found lacking at least one tumor marker, the majority (86%) were missing HER2. The exclusion of subjects without ER or PR results has been noted in other population-based cancer registry studies and our findings are similar [35-37]. Missing HER2 results have been described in our previous publications [27,28]. The St Gallen risk categories were applied retrospectively. Peritumoral vascular invasion was not recorded in the cancer registry abstract and could not be used for determination of the St Gallen risk categories. It is not entirely clear how this affects the risk categories. In a recent study, the adverse prognostic impact of peritumoral vascular invasion was limited to receptor-negative tumors regardless of chemotherapy [38]. Lastly, treatment information was minimal and generic in nature. Of the subjects known to have received some form of adjuvant therapy, the majority of ER-positive and ER-negative patients received endocrine therapy and chemotherapy, respectively. However, start and stop dates of treatments, and the specific forms of endocrine and chemotherapy were not available in the registry data. Anti-HER2 therapy was not recorded in the registry abstract. It should be noted that, in the United States, trastuzumab was approved for adjuvant treatment of breast cancer patients in November 2006, and thus the lack of anti-HER2 treatment data may have only a slight confounding effect. Lastly, patient accrual was from 2000 to 2006 and analyzed as of December 2008. We recognize that many patients were only at risk for two years and that many low risk patients may relapse after five years. Continued follow-up and analysis is planned.

Despite these shortcomings, we believe our study is of value because of the large number of breast cancer patients examined, reflecting real-world experience of a statewide cancer registry in an ethnically diverse population. We have shown how the use of ER/PR/HER2 subtype or phenotype expression highlights the marked heterogeneity of the Intermediate and High Risk categories of the 2007 St Gallen statements. We welcome the use of the molecular classification of breast cancer and gene-profiling assays. Further, we believe it is prudent to correlate molecular findings with less expensive techniques such as the use of ER/PR/HER2 subtypes and immunohistochemical (IHC) profiles, the so-called "Poor Man's IHC definitions of microarray-based intrinsic subtypes" [34].

## Conclusion

The use of ER/PR/HER2 subtype highlights the marked heterogeneity of the Intermediate and High Risk categories of the 2007 St Gallen statements. The use of ER/PR/HER2 subtypes and correlation with molecular classification of breast cancer is recommended.

## Competing interests

The authors declare that they have no competing interests.

## Authors' contributions

CP and VC conceived the idea for the study and interpreted the data. KB obtained the data from the California Cancer Registry and conducted the statistical analysis. All three authors contributed to the writing and have read and approved the final manuscript.

## Pre-publication history

The pre-publication history for this paper can be accessed here:

http://www.biomedcentral.com/1471-2407/10/228/prepub
